# Small molecule probes of protein aggregation

**DOI:** 10.1016/j.cbpa.2017.06.008

**Published:** 2017-06-22

**Authors:** Lydia M Young, Alison E Ashcroft, Sheena E Radford

**Affiliations:** Astbury Centre for Structural Molecular Biology, School of Molecular and Cellular Biology, Faculty of Biological Sciences, University of Leeds, Leeds LS2 9JT, UK

## Abstract

Understanding the mechanisms of amyloid formation and toxicity remain major challenges. Although substantial progress has been made in the development of methods able to identify the species formed during self-assembly and to describe the kinetic mechanisms of aggregation, the structure(s) of non-native species, including potentially toxic oligomers, remain elusive. Moreover, how fibrils contribute to disease remains unclear. Here we review recent advances in the development of small molecules and other reagents that are helping to define the mechanisms of protein aggregation in molecular detail. Such probes form a powerful platform with which to better define the mechanisms of structural conversion into amyloid fibrils and may provide the much-needed stepping stone for future development of successful therapeutic agents.

## Introduction

### Combatting amyloidosis

**Figure 1:**
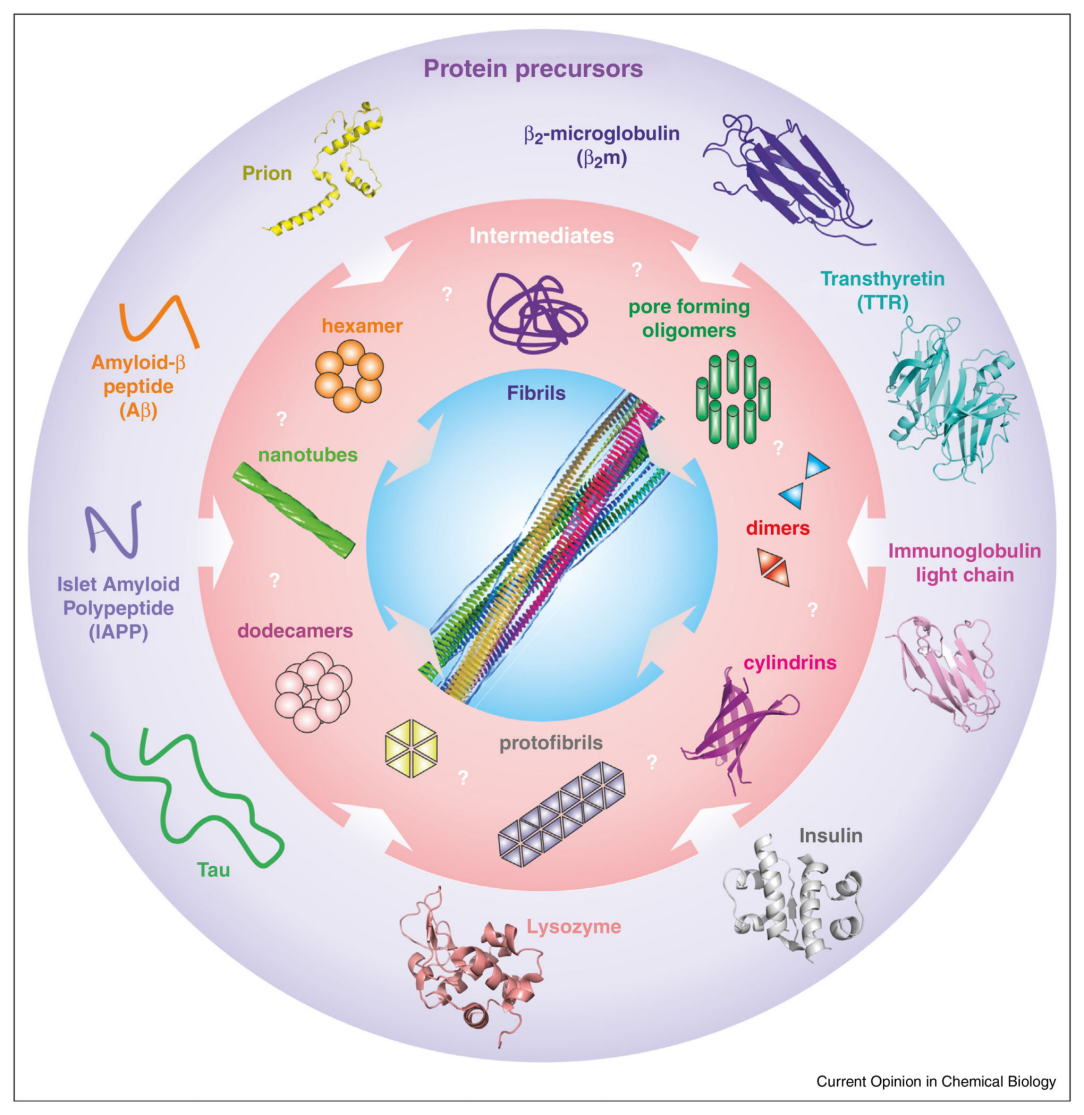
Sketches or structures, where available, of amyloid precursors and peptides associated with various human pathologies. PDB IDs: β_2_-microglobulin D76N, 4fxl [126], transthyretin, 3cn4 [127], immunoglobulin light chain, 1bre [128], insulin, 1zeh [129], lysozyme, 5fhw [130], human prion: 1i4m [131]. These unrelated amyloid sequences assemble into highly organised cross-β amyloid fibrils (centre) [132] via an array of oligomers that, thus far, have largely eluded structural characterisation. A number of possible oligomeric structures have been proposed [133,134], including dimers [135], domain swapped dimers [56], hexamers and dodecamers [136], cylindrins [12], nanotubes [137] and pore forming oligomers [133,134]. The structure of (insulin) fibrils shown in the centre is taken from [132], with permission.

An array of human diseases known collectively as ‘amyloidoses’ result from misfolding and aberrant assembly of proteins into amyloid fibrils (Figure 1) [1]. Amyloidoses are associated with the formation of extracellular amyloid fibrils and/or intracellular amyloid-like inclusions with a cross-b structure [1]. Amyloid diseases include neurode-generative disorders, such as Alzheimer’s, Parkinson’s, Creutzfeld-Jacob and Huntington’s disease (HD) [2–4]; non-neuropathic localised amyloidoses including type II diabetes mellitus (T2DM) [5], dialysis related amyloidosis [6] and familial amyloid neuropathy [7^•^]; and systemic amyloidoses such as light chain amyloidosis (AL) [8].

The structural and molecular mechanisms by which aggregation occurs, and how disease is triggered, remain elusive [9]. Current opinion points to pre-fibrillar oligomers as the major toxic species [10–12] (Figure 1), but mature amyloid fibrils can also be cytotoxic [13–16]. The array of oligomers formed by primary and secondary nucleation events [17] adds to the difficulty in isolating and identifying the toxic agents of amyloid disease. Moreover, oligomers are heterogeneous in mass, structure and stability [18^•^,^19^,^20^], and are in rapid exchange with each other, as well as with monomers and/or the fibrils themselves, making structural and functional characterisation of oligomeric species even more challenging [11,20–23]. As the toxic species in the majority of amyloidoses remain uncharacterised, available therapies focus on ameliorating symptoms [9,24,25], with reagents able to prevent, delay, or reverse amyloid disease (with one exception [26]) remaining beyond our grasp.

### Small molecules and other probes of protein aggregation

A number of small molecule probes able to track and/or inhibit protein aggregation have been developed recently. Some inhibit aggregation via direct interaction with the target protein [7^•^,^27^^••^,^28^], whilst others ameliorate aggregation by upregulating the cellular responses to the presence of aggregates [25,29,30]. Such ‘chemical chaperones’ can act by stabilising a protein’s ‘native’ structure, preventing misfolding and inhibiting protein self-assembly and its associated toxicity. Alternatively, by binding to the fibril surface, small molecules can disfavour secondary nucleation as a source of oligomer production [17,31^••^]. Small molecules have also been used to enhance the degradation of aggregates and may be used as a parallel, synergistic strategy to prevent aggregate-induced cytotoxicity [9,24]. Here we reflect on how these reagents are enabling new discoveries about the molecular mechanisms of protein aggregation and its associated cellular toxicity in amyloid disease.

#### β-Sheet breakers and peptidomimetics

Synthetic peptide derivatives, termed ‘β-sheet breakers’, have been developed that are able to inhibit amyloid formation by binding to monomeric precursors [32–35], or by preventing fibril elongation by blocking fibril ends [13,32,36]. Substitution of key residues in synthetic peptides corresponding to the amyloid core regions with prolines [37,38], or incorporating N-methyl modified amino acids, prevents hydrogen bond formation crucial to the cross-β structure [39^•^]. Clever use of these strategies within cyclic peptides has been used to create amyloid inhibitors and has enabled oligomeric intermediates to be isolated and characterised [40,41]. Recently, polymer–peptide conjugates have been shown to disassemble fibrils formed from the Alzheimer-related peptide Aβ_40_ through a direct interaction, opening the door to strategies to reduce fibril load [42].

#### Antibodies, nanobodies and chaperones

Another strategy to inhibit amyloid formation exploits the exquisite specificity and affinity of antibodies for their antigens. Accordingly, antibodies have been developed which bind to monomeric amyloid precursors and, thereby, inhibit aggregation [43–45]. Grafting the most aggregation-prone regions of amyloidogenic peptides into the Complementarity Determining Regions (CDR) of antibodies provides an alternative strategy able to retard aggregation of intrinsically disordered proteins (IDPs) [46–48,49^••^]. Despite enormous efforts, the use of antibodies as anti-amyloid agents has failed (thus far) in clinical trials, because of the induction of aberrant immune responses [50,51]. Antibody-based probes, including ‘nanobodies’ based on single-domain fragments of a camelid antibody [52–54], have also been used to trap amyloid intermediates, including those of β_2_-microglobulin (β_2_m) and human prion protein (PrP) [55,56]. Additionally, a small protein scaffold termed an ‘affibody’ [57] has been developed using a combination of rational design and protein engineering. This 6.5 kDa protein is able to bind Aβ_40_ with *K_d_* ~ 300 pM, preventing oligomerisation [58].

Molecular chaperones can also be used as anti-amyloid agents. Using elegant kinetic analyses Linse, Knowles and colleagues have shown that the molecular chaperone, BRICHOS, inhibits the catalytic cycle of oligomer formation of Aβ_42_ by binding to the fibril surface, and delays aggregation-associated toxicity in brain slices and mouse models [59]. The same group has also shown that a different chaperone, heat shock protein 70 (hsp70), prevents Aβ_42_ fibril elongation by capping fibril ends [60^••^], raising the possibility that simultaneous addition of multiple chaperones could have a synergistic effect. Other chaperone complexes have been discovered that are able to disaggregate fibrils [61–63], showing that amyloid deposition can be reversed despite the enormous thermodynamic stability of the cross-β fold [64,65]. Together, these results point to an exciting future for the use of natural, designed, or engineered proteins to interrogate the molecular mechanisms of aggregation and, potentially, to control disease onset and/or the progression of disease.

#### Small molecules able to control amyloid formation

Small molecules have several advantages over peptide-based and antibody-based strategies, including the higher stability of small molecules in biological fluids and tissues, their potential to cross the blood–brain barrier, and their immunological tolerance [66]. Although structure-based design can be used, in principle, to create small molecules able to stabilise proteins that form amyloid from an initially folded structure [67], many amyloid precursors are IDPs (Figure 1), precluding structure-based drug design. Indeed, it was initially purported that IDPs would be ‘undruggable’, given the unfavourable entropy change that would result from binding-induced folding [48,68,69]. Excitingly, this has now been shown not to be the case, with recent examples including inhibition of Aβ_40/42_ (Alzheimer’s), islet-associated polypeptide (IAPP) (type II diabetes mellitus), or α-synuclein (Parkinson’s) by small molecules [23,27^••^,^28^,^70^^••^,^71^^••^,^72^,^73^]. Indeed, entropic stabilisation of IDPs (brought about by local folding of an IDP upon ligand binding with enhancement of conformational flexibility elsewhere), suggests that small molecules may be able to bind many IDPs with sufficient affinity to inhibit amyloid formation [74^•^].

#### Polyphenols: commonly used inhibitors of amyloid, but a PAIN

Initial successes in the development of small molecule inhibitors of amyloid formation were inspired by the observation that Thioflavin-T (ThT) and Congo red (histological stains for amyloid) inhibit amyloid assembly of several proteins, including Aβ_40/42_ and IAPP when present at sufficiently high concentrations [75–78]. Over the past two decades, a panoply of small molecule inhibitors of amyloid formation has been reported, the most common family of which are polyphenols [79,80,75]. Most common of all, the flavanol epigallocatechin gallate (EGCG), has been shown to prevent oligomerisation and fibril formation, and to promote disaggregation of preformed fibrils in multiple amyloid systems, including Aβ_40_, Aβ_42_, IAPP and α-synuclein, both *in vitro* and *in vivo* [23,75,81–83]. A disadvantage of polyphenol compounds as aggregation inhibitors, however, is their lack of specificity [84–87]. Indeed, soluble and planar aromatic compounds, which are often abundant in small molecule libraries, have potential to act as β-sheet intercalators, and hence may be erroneously identified as anti-amyloid therapeutics in screens. In addition, polyphenols are pan-assay interference compounds (PAINS), that is, small molecules that result in false positives in screens for small molecule ligands as a result of promiscuous binding, covalent modification of the target, small molecule reactivity, aggregation of the small molecule itself, or interference with fluorescence-monitored assays [88^•^,^89^]. These ‘chemical con artists’ include curcumin, previously hailed as a potent and generic anti-amyloid agent [88^•^]. Furthermore, molecules that delay aggregation by increasing the lifetime of oligomeric species and/or promoting fibril depolymerisation could be dangerous if they also increase the population of potentially toxic oligomers. The search for small molecule inhibitors should possibly shift, therefore, in favour of molecules that bind to the monomeric precursor and prevent the formation of oligomers, avoiding the need to identify a specific oligomeric species as a target. Small molecules that retard secondary nucleation, fibril depolymerisation and/or oligomer ‘shedding’ by binding to the fibril itself are an exciting alternative strategy [11,20,90].

### To screen or to design? That is the question

Discovery of aggregation inhibitors for amyloid precursors which lack a well-defined structure, has been restricted to screening compound libraries using biophysical techniques or dye binding assays [91^•^,^92^–^94^]. Below we highlight four new and complementary strategies that have been used to identify small molecule inhibitors of IAPP and/or Aβ_40_ aggregation. Although causing different diseases (type II diabetes mellitus and Alzheimer’s, respectively), these 37 and 40 residue IDPs share 47% sequence similarity. They are also able to co-assemble *in vitro* and are even found co-localised in plaques in Alzheimer brains [19,95].

#### Screening for anti-aggregation inhibitors in the periplasm of E. coli

**Figure 2:**
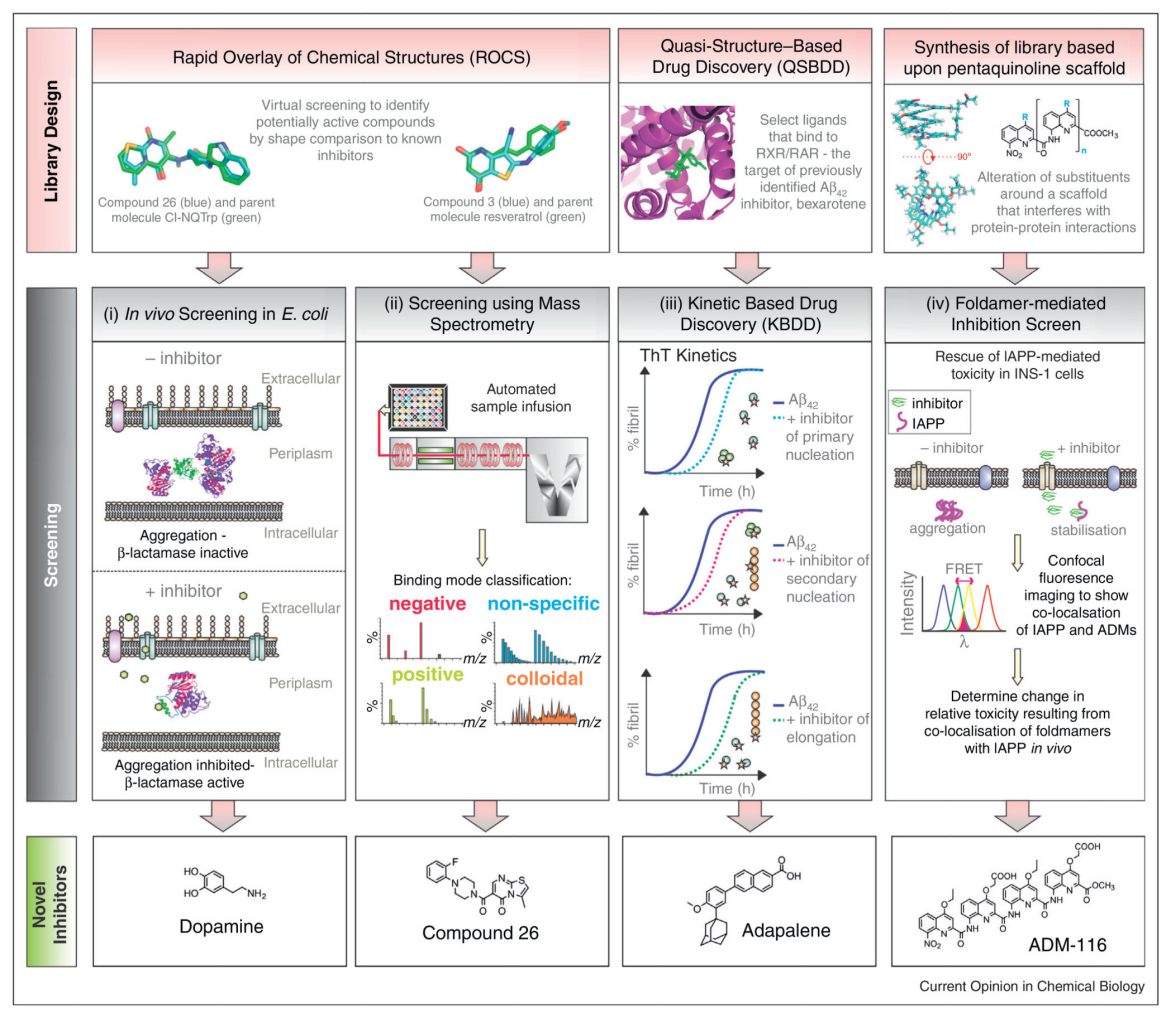
Schematic representing different strategies to screen for small molecule inhibitors of aggregation. The methods developed include (I) the expression of the target protein (green) within a split β-lactamase construct (purple and pink) in the *E. coli* periplasm. Small molecules (<600 Da) (green hexagon) are able to diffuse freely through porins in the outer membrane, enabling antibiotic resistance to be used as a readout for molecules able to prevent aggregation [27^••^]. Using this screen, dopamine (structure shown) was found to prevent IAPP aggregation. Figure adapted from Ref. [27^••^]. (II) Screening of potential ‘hits’ using native ESI-MS, in combination with ion mobility spectrometry (IMS), enables the mode and specificity of small molecule binding and the effect on oligomer distributions to be determined directly [70^••^]. Figure adapted from Refs. [28,70^••^]. Using Rapid Overlay of Chemical Structures (ROCS) and the known inhibitors of Aβ_40_ aggregation, chloronaphthoquinine tryptophan (Cl-NQTrp) or resveratrol (green) as templates (upper image) led to the discovery of new inhibitors (blue) of IAPP and/or Aβ_40_ aggregation (e.g. compound 26 (lower)) [70^••^]. (III) Quasi structure based drug design inspired by screening of orphan drug libraries for initial ‘hits’, combined with detailed kinetic analysis, enabled the mechanism of action of small molecule inhibitors of Aβ_42_ aggregation to be deduced [72]. The structure of adapalene, one of the most effective molecules identified, is shown. This compound delayed the aggregation of Aβ_42_ > three-fold when present at a 0.5 molar equivalent concentration with respect to Aβ_42_ [72]. (IV) The proposal that IAPP (and Aβ) aggregate through membrane-bound helical intermediates inspired the development of helical foldamers as potential anti-amyloid reagents [71^••^,^105^,^115^]. A synthetic tetraquinoline, ADM-116 (structure shown), is described that docks specifically with, and stabilises an α-helical intermediate of, IAPP and subsequently rescues β-cells from toxicity [71^••^].

Screening for small molecule inhibitors using *in vivo* assays averts some of the caveats of screening *in vitro*, including the requirement for large amounts of purified aggregation-prone protein and the use of unreliable dyebinding assays [96,97]. By adapting a method used previously to screen for mutations enhancing protein stability [98], a split β-lactamase host−guest system has been used to screen for small molecules able to decrease aggregation of IAPP using enhanced antibiotic resistance as the readout (Figure 2(I)) [27^••^]. The system is able to distinguish aggregation-prone from less aggregation-prone sequences (including human/rat IAPP and Aβ_42_/Aβ_40_), as well as to screen for aggregation inhibitors of each protein. Since molecules <600 Da can diffuse freely into the periplasm via porins in the outer membrane, the β-lactamase host–guest system is ideally placed to screen for ligands able to prevent aggregation. As the periplasm is oxidising, amyloid precursors containing disulphide bonds can also be assayed, offering advantages over screens in the cytoplasm [99^••^,^100^].

#### Identifying the targets of inhibition using non-covalent mass spectrometry

A key challenge in developing anti-amyloid reagents is to identify the species to which an inhibitor binds. For this purpose, electrospray ionisation-ion-mobility spectrometry-mass spectrometry (ESI-IMS-MS) is ideal [23,28,70^••^,^101^,^102^]. Screening ligand binding by ESI-IMS-MS enables rapid identification of the binding mode (specific, non-specific or colloidal (Figure 2(II)); the individual species with which the inhibitor interacts; and the effect of binding on oligomer formation [28,70^••^]. ESI-IMS-MS is also rapid (<1 min/sample), consumes low amounts of sample (~1000 molecules can be screened/mg protein), does not require sample labelling or immobilisation, provides stoichiometric and conformer-specific information, and readily identifies colloidal inhibitors that may be erroneously classified as ‘hits’ in other assays. ESI-MS is also ideal to detect PAINS compounds since the mass of both protein and ligand are directly determined [27^••^,^70^^••^]. Combining the split β-lactamase system [27^••^] with the ESI-IMS-MS-based screen [70^••^] led to the identification of small molecules that bind IAPP and/or Aβ_40_ and inhibit aggregation *in vitro* and in the bacterial periplasm (Figure 2(I) and (II)).

#### Quasi-structure-based drug discovery (QSBDD)

Amyloid assembly reactions typically display sigmoidal growth curves which consist of a ‘lag phase’, ‘elongation phase’ and, finally, a ‘plateau phase’ in which the system reaches equilibrium and monomer concentration remains constant [17]. Kinetic analyses have shown that these phases cannot be ascribed to a single microscopic process, but each phase is instead the amalgamation of multiple concomitant processes including fibril elongation, fragmentation and/or secondary nucleation [17,31^••^,^60^^••^] (Figure 2(III)). Given that the toxic species in amyloid disease remain elusive, it is vital that the effect of an inhibitor on the growth profile, and hence on the population of potentially toxic oligomeric species, is known. Indeed, lengthening of the ‘lag phase’ via the delay of elongation could increase the population of toxic species and exacerbate disease [10,103,104].

Using quantitative chemical kinetics, Vendruscolo, Knowles, Dobson and co-workers have determined the effect of small molecules on specific microscopic events in the aggregation of several proteins, including Aβ_42_ [72]. Although this approach does not enable the species that bind the ligand to be identified, which is possible using ESI-MS, chemical kinetics enable analysis of how oligomer population, fibril yield, fibril fragmentation and secondary nucleation are affected by ligand binding. Using ThT kinetics as the assay and a library of orphan drugs, the clinically approved anti-cancer drug bexarotene — an agonist of the retinoid X receptor (RXR) — was identified as a potent inhibitor of Aβ_42_ aggregation and its mechanism of action ascribed to the prevention of primary nucleation [104]. Building on this study, the authors then utilised a ‘quasi-structure-based drug discovery’ (QSBDD) strategy (Figure 2(III)) in which twelve other known ligands of the RXR (and retinoid A receptor (RAR)) were screened for their ability to inhibit Aβ_42_ aggregation [72]. The rate constants for aggregate growth in the presence of the inhibitors were used to identify the microscopic process with which the compounds interfere [72]. These experiments showed that the more modest inhibitors of Aβ_42_ aggregation inhibit primary nucleation, whilst more potent inhibitors retard all three major steps in Aβ_42_ aggregation (primary nucleation, elongation and surface-catalysed secondary nucleation). The potent inhibitors were also shown to rescue Aβ_42_-mediated toxicity in *Caenorhabditis elegans*, demonstrating the potential of the kinetics-based assay for drug discovery.

#### Foldamer inhibitors of amyloid formation

Exploiting the foldedness of a small molecule, Hamilton, Miranker and co-workers have developed ‘synthetic foldamers’ (Figure 2(IV)) as amyloid inhibitors of IAPP and Aβ_42_ [71^••^,^105^–^107^]. A foldamer occupies a specific structure in the absence of a binding partner as a result of the inherent conformational preferences of its subunits [108–110]. Owing to the diversity of backbones and functional groups and their virtually unlimited geometries, foldamers present a vast array of molecular architectures with the potential to target a wide range of biomolecules in a sequence-specific and structure-specific manner [110]. Indeed, both peptidic and polyaromatic foldamers have been developed that are capable of cell penetration [110] and specific binding to a variety of biomolecular targets, including carbohydrates [111], membranes [112], proteins [71^••^,^105^] and RNA molecules [113] with μM affinity [110].

Hamilton and Miranker first described the use of oligomeric pyridylamides as IAPP aggregation inhibitors, with a pentameric foldamer binding to IAPP with a *K_d_* of 40 μM and inhibiting fibril assembly [114,115]. Building on these studies, these authors recently described the development of a novel tetraquinoline amide foldamer (Figure 2(IV) (top)), named ADM-116 [71^••^]. This compound is able to cross the plasma membrane, recognise its target (IAPP), stabilise α-helical conformers of this IDP, and prevent IAPP-induced cytotoxicity in INS-1 cells [71^••^]. These studies pave the way for the use of foldamers as aggregation inhibitors and, perhaps most excitingly, to determine whether toxicity is associated with extracellular and/or intracellular events for these protein precursors [10,71^••^].

### Future perspectives

Probing the mechanisms of amyloid assembly and alleviating toxicity remain enormous challenges. The dynamic and short-lived nature of non-native monomers and oligomers and the polymorphism of fibrils themselves [116] will require ingenious experimental and theoretical approaches to reveal how and why proteins aggregate into amyloid and cause disease. As described above substantial progress has been made recently in our ability to interrogate amyloid assembly mechanisms through kinetic analyses [17] and experiments that enable individual species to be tracked during assembly [23,70^••^,^117^,^118^]. Although ‘toxic’ oligomers seem the most obvious therapeutic target, their heterogeneity, in terms of structure, stability and toxicity, makes ligand discovery challenging and suggests that the best strategy may be to administer several compounds in combination. These may block binding sites to prevent further assembly or to render oligomers/fibrils less toxic. Blocking early events in amyloid assembly, such as rarely populated monomeric conformers or the monomer to dimer transition, using small molecules is a significant challenge given the disordered/partially folded nature of amyloid precursors [48,68] and the weak binding constants of many aggregation inhibitors [66]. Despite significant break-throughs in screening methods and the ability to determine the conformational properties of small oligomers using techniques such as ESI-IMS-MS as described above [28,70^••^], whether small molecules that have promising activities *in vitro* will be active within the complex milieu of a cell or a living organism remain additional unknowns. Together, these factors and the lack of attention to the complex mechanisms underlying amyloid disease [119] have led to the failure of many clinical trials [120,121]. These failures have contributed to amyloid proteins being questioned as therapeutic targets, despite their obvious importance.

Despite these challenges the future looks bright. Our understanding of the effects of protein aggregation on cellular function has increased, with new understandings of how aggregates are recognised by molecular chaperones, transported into different organelles, and targeted for degradation [122,123]. Chaperone complexes able to disaggregate fibrils have also been discovered [61,124]. This armoury of information may enable small molecules able to target specific species, or specific phases of aggregation, to be developed using combinations of small molecule screening and/or design. These reagents should then provide an answer to the key questions that have exercised amyloid researchers for more than a century: which are the toxic species and how is toxicity manifested in cells?

Despite the common cross-β structure of amyloid, different amyloidogenic proteins may disrupt cellular homeostasis through different mechanisms [88]. Hence, one therapeutic strategy may not be appropriate for all amyloid diseases. Since the rates of aggregation are highly dependent on protein concentration, even small changes in the population of an amyloid precursor could have a profound effect on disease. Accordingly, small molecules that bring about only minor changes in aggregation rate could be highly beneficial. Combining small molecules which upregulate proteostasis mechanisms with ligands that target the aggregation precursor could be the most powerful strategy, as exemplified by such a combined therapeutic strategy for lysosomal storage diseases [125]. Given that amyloid fibrils can play a functional role in bacteria and fungi [88], anti-amyloid agents could also be useful as anti-microbials. In both scenarios, small molecules will play an important role in enhancing our fundamental understanding of amyloid formation mechanisms and in their exploitation to combat disease.
